# Instructor Development Workshops for Advanced Life Support Training Courses Held in a Fully Virtual Space: Observational Study

**DOI:** 10.2196/38952

**Published:** 2022-06-29

**Authors:** Tetsuro Kiyozumi, Norio Ishigami, Daisuke Tatsushima, Yoshiyuki Araki, Yuya Yoshimura, Daizoh Saitoh

**Affiliations:** 1 Department of Defense Medicine National Defense Medical College Tokorozawa Japan; 2 Department of Emergency and Critical Care Medicine Hachinohe City Hospital Hchinohe Japan; 3 Division of Traumatology, Research Institute Department of Traumatology and Critical Care National Defense Medical College Tokorozawa Japan

**Keywords:** virtual reality, virtual space, instructor development workshop, resuscitation training course, advanced life support, resuscitation training, digital training, virtual learning, digital education, medical education

## Abstract

**Background:**

Various face-to-face training opportunities have been lost due to the COVID-19 pandemic. Instructor development workshops for advanced resuscitation (ie, advanced life support) training courses are no exception. Virtual reality (VR) is an attractive strategy for remote training. However, to our knowledge, there are no reports of resuscitation instructor training programs being held in a virtual space.

**Objective:**

This study aimed to investigate the learning effects of an instructor development workshop that was conducted in a virtual space.

**Methods:**

In this observational study, we created a virtual workshop space by using NEUTRANS (Synamon Inc)—a commercial VR collaboration service. The instructor development workshop for the advanced life support training course was held in a virtual space (ie, termed *the VR course*) as a certified workshop by the Japanese Association of Acute Medicine. We asked 13 instructor candidates (students) who participated in the VR course to provide a workshop report (VR group). Reports from a previously held face-to-face workshop (ie, the face-to-face course and group) were likewise prepared for comparison. A total of 5 certified instructor trainers viewed and scored the reports on a 5-point Likert scale.

**Results:**

All students completed the VR course without any problems and received certificates of completion. The scores for the VR group and the face-to-face group did not differ at the level of statistical significance (median 3.8, IQR 3.8-4.0 and median 4.2, IQR 3.9-4.2, respectively; *P*=.41).

**Conclusions:**

We successfully conducted an instructor development workshop in a virtual space. The degree of learning in the virtual workshop was the same as that in the face-to-face workshop.

## Introduction

### Background

Excellent instructors are indispensable for conducting advanced resuscitation (ie, advanced life support [ALS]) training courses, and there are existing instructor training programs for ALS training courses that are accredited by academic societies and other organizations. However, due to the COVID-19 pandemic, various face-to-face training opportunities have been lost [1-3], and instructor training programs are no exception. The use of remote meeting systems that use 2D screens is a common method of remote training. However, it is difficult to apply this methodology to a hands-on training course in which tools are used in a 3D space. Remote training using virtual reality (VR) is an attractive strategy for conducting experiential training without the need to gather people together [4-7]. Real-time, interactive interactions in a variety of situations that unfold in a virtual space can be expected to enhance the learning process [4] and improve communication skills, logical thinking, and decision-making skills [5]. The experiences in a fully immersive environment enhance learning, resulting in high knowledge retention and the development of empathy [6]. However, VR content still does not have enough tactile fidelity [1], skill acquisition via VR training may be inferior to skill acquisition via face-to-face training, and there are technical difficulties with conducting practical skills training by using advanced resuscitation equipment within a completely virtual space [8,9].

### Importance

In terms of instructor training, there is no need to faithfully reproduce exact real-life skills in all courses. For example, conventional face-to-face instructor training programs have also been developed with an emphasis on teaching and facilitation techniques, often omitting the details of skills related to advanced resuscitation. Therefore, it is conceivable that the necessary learning effect could be obtained even when instructor training programs are held in a virtual space. However, few studies have evaluated the implementation of nontechnical skill building related to educational techniques in virtual spaces [10]. We did not find any reports of resuscitation instructor training programs that were held in a virtual space.

### Goals of This Study

This study aimed to investigate the learning effects of an instructor development workshop for an immediate cardiac life support (ICLS) course—an ALS training course that was approved by the Japanese Association of Acute Medicine (JAAM) [11] and conducted in a virtual space.

## Methods

### Study Design and Setting

In this observational study, we created a virtual workshop space within NEUTRANS (Synamon Inc) [12]—a VR collaboration service. NEUTRANS is a commercial service that allows users to interact as avatars in a virtual space by using a head-mounted display. Users are able to walk around freely, grab objects, and otherwise navigate within the virtual space. This service also allows users to present presentation materials, view 360° videos, and use whiteboards and memos. It is possible to hold lectures, group discussions, practical training sessions, and role-plays in this virtual space.

**Figure 1:**
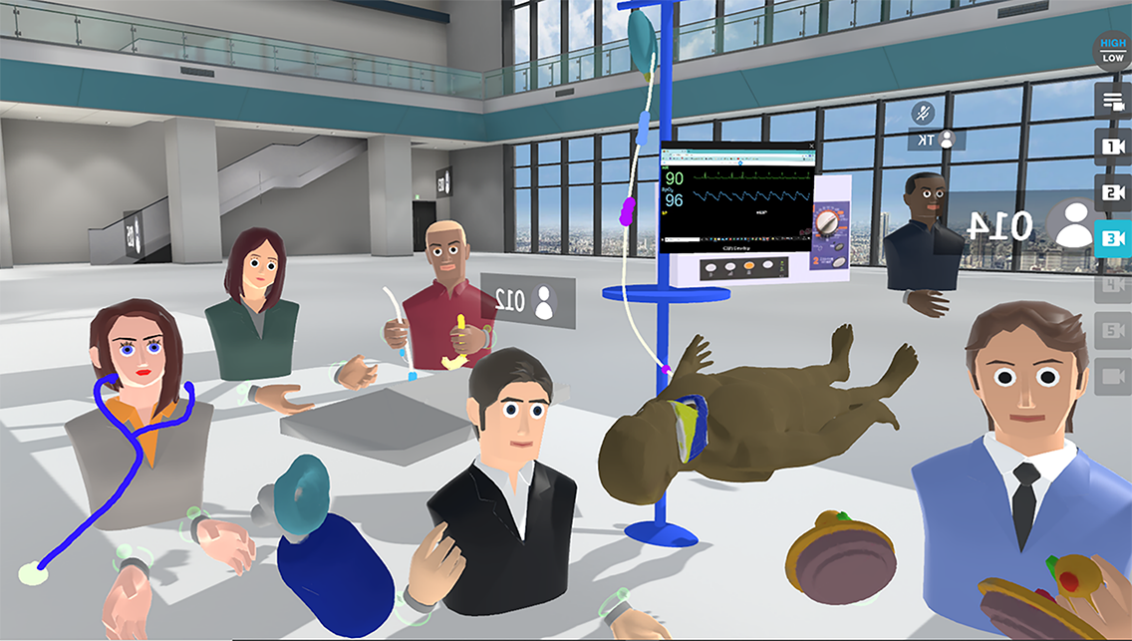
Scenery in the virtual space. We simulated an advanced resuscitation training course in a virtual space. The training mannequin and resuscitation equipment were created by using 3D models. The participants acted as avatars in the virtual space, and the patient monitor could be operated in a virtual section of the space.

In this study, we did not have expertise in programming or computer graphics, but we designed 3D models of dolls and resuscitation equipment by using the *paint* function in NEUTRANS, Oculus Medium (Adobe Inc), and the Windows 10 (Microsoft Corporation) onboard *paint* function. We placed these models in the virtual space for practical training sessions and role-plays. In addition, the simulated patient monitoring system—EmerSim (Penguin System Co, Ltd)—was reflected in the virtual space by using the *desktop sharing* function. This enabled the operation and display of the simulated patient monitor in virtual space (Figure 1).

We developed the program curriculum in accordance with the standard workshop program, which was accredited by the JAAM (Table 1), and held a trial workshop in March 2021. Instructor trainers and instructor candidates (students) entered the virtual space as avatars by using the Oculus Quest 2 (Meta Platforms Inc) or Oculus Lift (Meta Platforms Inc) and participated in the workshop. The status of the trial workshop was presented to the JAAM ICLS committee. After December 25, 2021, the workshop in the virtual space was accredited as an official course.

**Table 1:** Program for the instructor development workshop.

Time	Agenda	Type	Notes
8:30 AM to 9 AM	Opening	Presentation and PT^a^	Included VR^b^ equipment operation practice
9 AM to 9:40 AM	What is instruction?	SGD^c^	After discussing ideal instruction methodology, each group will present their ideas
9:40 AM to 10 AM	Education techniques	Presentation	Instructional procedures, nonverbal communication, feedback, facilitation, and coaching
10:15 AM to 10:45 AM	Explaining the use of supplies	RP^d^	As an instructor, describe the use of supplies (bag masks, scissors, etc) to 1 student
11 AM to noon	Teaching skills	RP	As an instructor, teach skills (ventilation, use of an AED^e^, etc) to several students
1 PM to 1:30 PM	Setting up a simulation booth	PT	Trial an ideal arrangement of teaching materials (mannequins and resuscitation equipment)
1:45 PM to 2:45 PM	Performance evaluation and feedback	RP	Watching a 360° video of a resuscitation team in action, scoring performance by using a checklist, and providing feedback
3 PM to 4:30 PM	Leading a scenario exercise	RP	Act as an instructor to facilitate a scenario exercise until the time of posttraining feedback
4:30 PM to 5 PM	Summary	SGD and presentation	Discuss and present ideal instruction methods and award certificates of completion

^a^PT: practical training.

^b^VR: virtual reality.

^c^SGD: small group discussion.

^d^RP: role-play.

^e^AED: automated external defibrillator.

### Intervention and Outcomes

We held the evaluated workshop in a virtual space (ie, the VR course) as a certified course in December 2021 and February 2022. Invited, voluntary participants were enrolled in this workshop. A total of 13 instructor candidates (students) who participated in the VR course (ie, the VR group) were asked to provide a workshop report. The reports from a face-to-face workshop (ie, the face-to-face course) that was held in June 2021 (ie, the face-to-face group) were prepared for comparative evaluations.

A total of 5 certified ICLS workshop instructor trainers viewed and scored each element of the reports on a 5-point Likert scale (poor=1; excellent=5) to evaluate the overall evaluation score. The instructor trainers also rated the reports on a 5-point Likert scale regarding whether they thought a report was a face-to-face course report or a VR course report (ie, the “VR-like score”). We analyzed the characteristics of the workshop reports by using text mining techniques.

After the VR course, we asked 13 students and 7 instructor trainers who participated in the VR course to rate their satisfaction with the course, the operability of the VR equipment, the occurrence of VR sickness, and whether each agenda item was judged to be suitable for implementation in VR. These items were included in a questionnaire, presented to the participants, and evaluated by using a 5-point Likert scale.

### Statistical Analysis

We examined the scores’ interrater agreement by using the Kendall agreement coefficient. Following this, we used the average of the five raters' scores as the score for each student. We performed between-group comparisons of scores by using the Mann-Whitney *U* test.

We analyzed the questionnaire findings by using the Mann-Whitney *U* test, Friedman test, or Bonferroni multiple comparison test, as appropriate. The primary end point of this study was the overall evaluation score. *P* values of <.05 were considered statistically significant. All statistical analyses were conducted by using R statistical software (R Foundation for Statistical Computing) [13]. We performed a textual analysis by using KH Coder (Iknow LLC) [14,15].

### Ethics Approval

This study was approved by the Ethics Committee of the National Defense Medical College (reference number: 4488) and was conducted with the consent of all participants. Moreover, we conducted this study in accordance with the principles of the Declaration of Helsinki and its later amendments.

## Results

### Study Participant Characteristics

Participants completed the VR course without any problems, and all students received certificates of completion. Participants in the December 2021 course logged in from 2 laboratories on the campus of the National Defense Medical College.

In the February 2022 course, participants logged in from 5 locations—3 laboratories on the campus of the National Defense Medical College, a meeting room in Hachinohe City Hospital (located approximately 600 km from the National Defense Medical College), and a private home. More specifically, the participant who signed in from a private home was affiliated with one of the laboratories and was identified as a person who had contact with a patient with COVID-19 (ie, 2 days prior to the course); thus, we placed this participant on home standby, which resulted in hastily arranged participation.

### Main Results

We collected workshop reports from 13 students who were enrolled in the VR course (the VR group) and 10 students enrolled in the face-to-face course (the face-to-face group) to conduct comparative evaluations (report recovery rate: 23/23, 100%).

**Figure 2:**
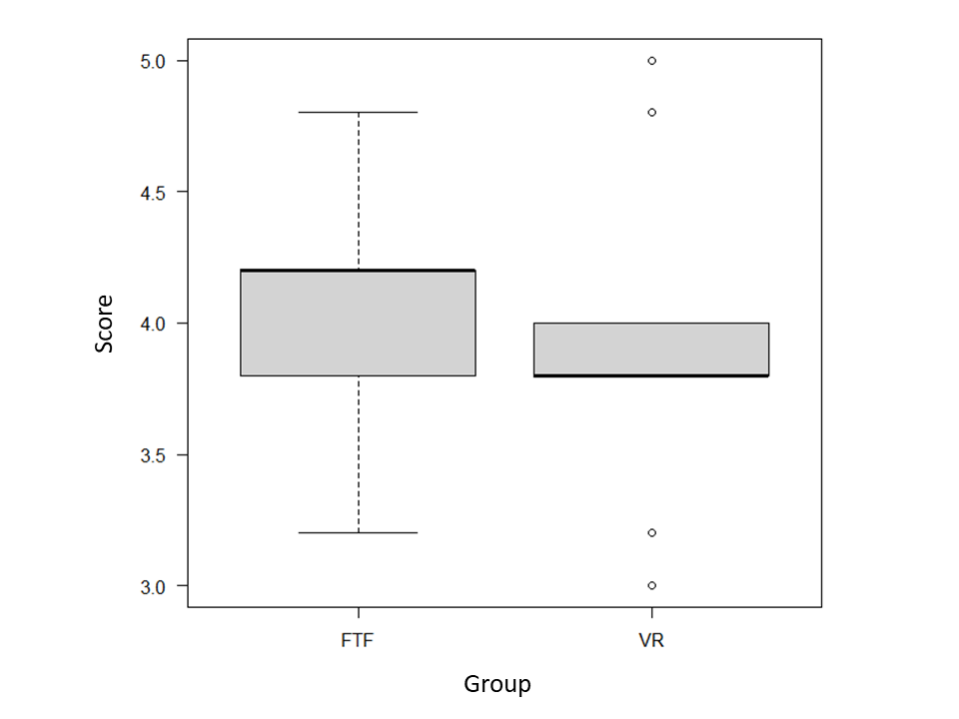
Overall evaluation scores. The reports were scored on a 5-point Likert scale (1=poor; 5=excellent), and the averages from the evaluations conducted by the five instructor trainers were analyzed as the overall evaluation score. The black line indicates the median, the gray box indicates the range between the 25th and 75th percentiles, and the minimum and maximum values are indicated by lines or white circles. The overall evaluation scores for the VR and FTF groups did not differ at the level of statistical significance (median 3.8, IQR 3.8-4.0 and median 4.2, IQR 3.9-4.2, respectively). No statistically significant differences were observed within the Mann-Whitney U test results (*P*=.41). FTF: face-to-face; VR: virtual reality.

**Figure 3:**
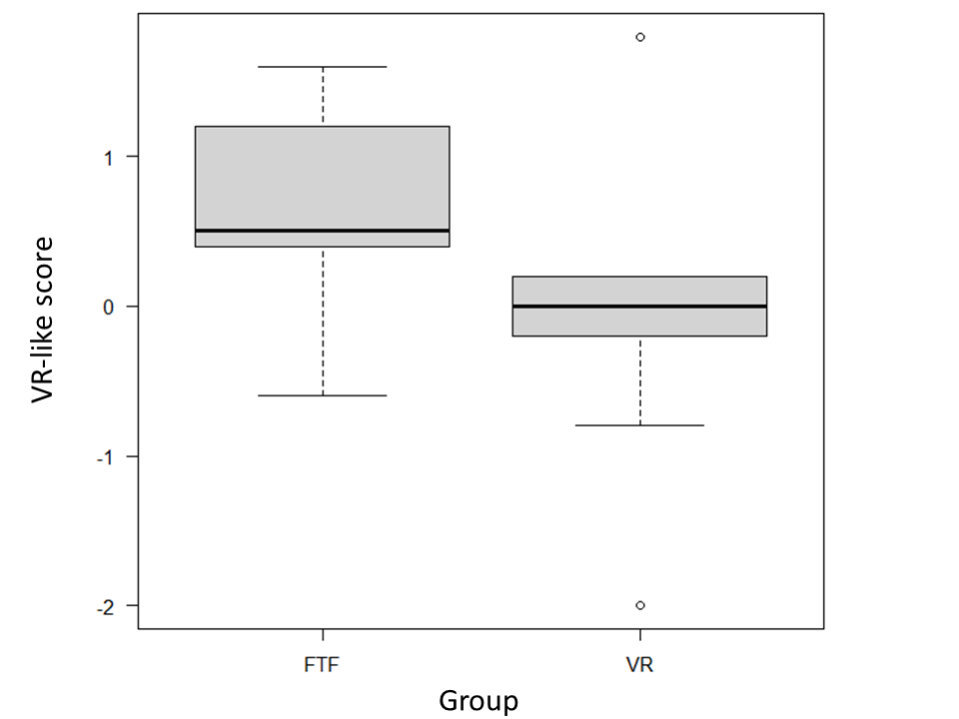
VR-like scores. A total of 5 instructor trainers rated the reports on a 5-point Likert scale regarding whether they thought a was an FTF course report or a VR course report (VR=–2; FTF=2), and the averages were analyzed as “VR-like scores.” The black line indicates the median, the gray box indicates the range between the 25th and 75th percentiles, and the minimum and maximum values are indicated by lines or white circles. The median VR-like scores for the VR group and the FTF group were 0.0 (–0.2 to 0.2) and 0.5 (0.4-1.1), respectively. The results for the FTF group were rated as more “FTF-like” according to the findings of the Mann-Whitney U test (*P*=.03). FTF: face-to-face workshop; VR: virtual reality.

The students’ characteristics are listed in Table 2. With regard to evaluations of the workshop reports, the interrater agreement for the five certified instructor trainers was as follows: overall evaluation score=0.71 and VR-like score=0.77 for the Kendall agreement coefficient. The overall evaluation scores for the VR and face-to-face groups did not differ at the level of statistical significance (median 3.8, IQR 3.8-4.0 and median 4.2, IQR 3.9-4.2, respectively; *P*=.41; Figure 2). The median VR-like scores for the VR group and the face-to-face group were 0 (IQR −0.2 to 0.2) and 0.5 (IQR 0.4-1.1), respectively (Figure 3). We rated the face-to-face group as more “face-to-face–like,” and these findings were statistically significant (*P*=.03).

**Figure 4:**
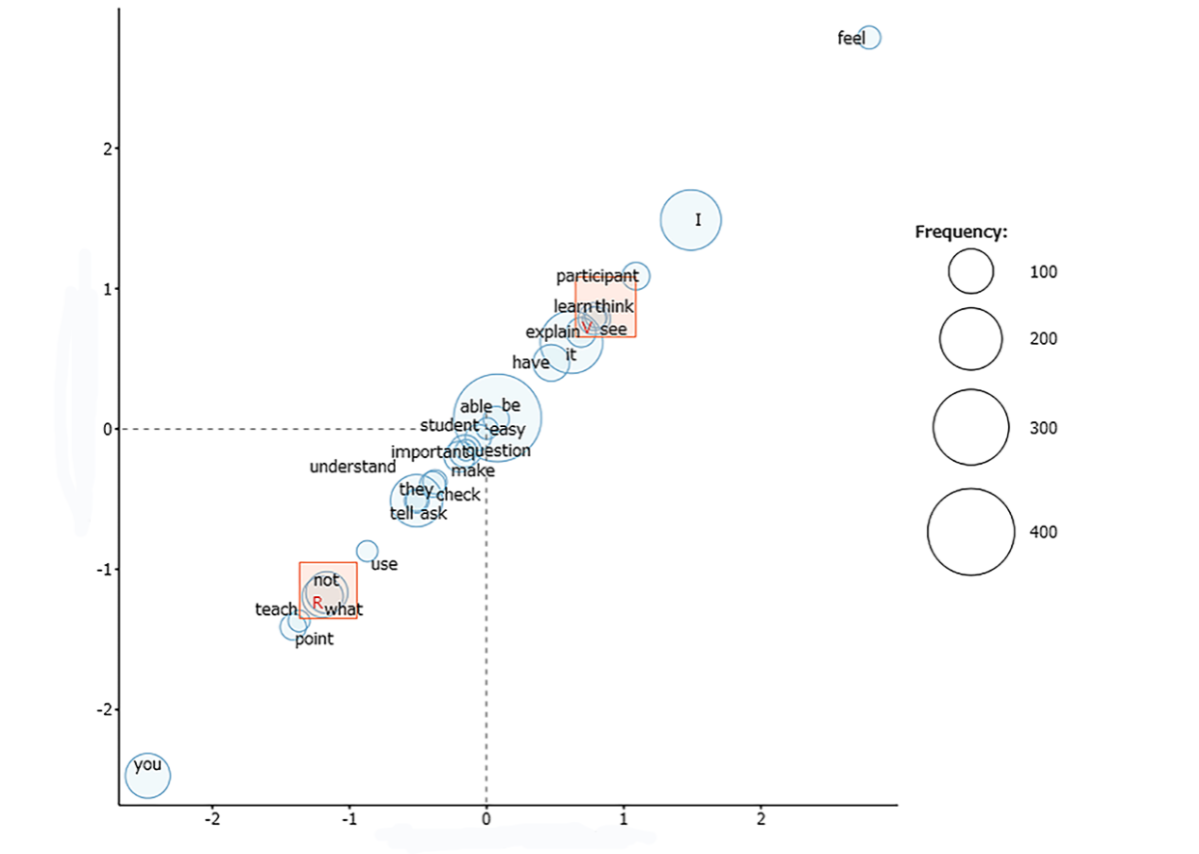
Textual analysis of the workshop reports. The "V" in the square represents the coordinates of the virtual reality (VR) group, and the "R" in the square represents the coordinates of the face-to-face (FTF) group. The size of the circles indicate the frequency of the words in the reports. The circles close to the reference point (0, 0) represent words that are not characteristic of the group, whereas the circles closer to the coordinates of each group represent words that are more characteristic of that group. The VR group was characterized by the words “feel,” “I,” “participant,” and “think,” while the FTF group was characterized by the words “you,” “points,” and “teach.”.

The results of the textual analysis are shown in Figure 4. The words *feel*, *I*, *participant*, and *think* characterized the VR group, and the words *you*, *points*, and *teach* characterized the face-to-face group.

We received questionnaires from 8 students and 6 instructor trainers who attended the VR course (report recovery rate: 14/20, 70%). The results are presented in Table 3. All respondents (14/14, 100%) were satisfied with the VR course, providing a score of 4 or higher on the 5-point Likert scale. Although some found the operation of the VR equipment to be confusing, this did not interfere with the progress of the course. Everyone participated until the end of the course, although some participants experienced VR sickness.

**Table 2:** Descriptive characteristics of the enrolled students.

Characteristics	Virtual reality group (n=13)	Face-to-face group (n=10)
Residents, n	1	1
Medical students, n	0	1
Nursing students, n	5	4
Nurses, n	5	3
Technicians (EMTs^a^ and RTs^b^), n	2	1
Age (years), mean (SD)	29.1 (7.6)	26.1 (6.4)
Ratio of men to women	4:9	3:7

^a^EMT: emergency medical technician.

^b^RT: radiology technician.

**Table 3:** Questionnaire results.

		Instructor trainers	Students
Sample size, n		6	8
Course satisfaction^a^, score (median [IQR])		5 (4.25-5)	4.5 (4-5)
Operability of virtual reality equipment^a^, score (median [IQR])		4 (4-4)	4 (3-4)
Virtual reality sickness^b^, score (median [IQR])		5 (4.25-5)	5 (4.50-5)
**Suitability for implementation in virtual reality^c^, score (median [IQR])**			
	Discussion: what is instruction?	5 (3.5-5)	5 (3-5)
	Presentation: education techniques	3 (3-4.5)	5 (3-5)
	Role-play: explaining the use of supplies	5 (3.5-5)	5 (4.5-5)
	Role-play: teaching specific skills	3 (3-4.5)	3 (1-3.5)
	Practical training: setting up a simulation booth	3 (3-4.5)	3 (3-5)
	Role-play: performance evaluation and feedback	5 (5-5)	5 (3-5)
	Role-play: leading a scenario exercise	4 (3-5)	5 (1-5)

^a^Likert scale: bad=1; good=5.

^b^Likert scale: severe=1; none=5.

^c^Likert scale (suitable for implementation in virtual reality): no=1; yes=5.

There was a statistically significant difference (*P*=.03) between responses regarding the suitability of VR implementation, depending on the agenda of the workshop. Although we did not detect statistically significant differences in comparative evaluations between each agenda item, many respondents indicated that the teaching of specific discrete skills was not suitable for VR training, whereas the domains of performance evaluation and feedback were deemed suitable for VR training. There were no statistically significant differences in responses between the students and the instructor trainers (what is instruction: *P*=.94; education techniques: *P*=.33; explaining the use of supplies: *P*=.75; teaching specific skills: *P*=.54; setting up a simulation booth: *P*=.89; performance evaluation and feedback: *P*=0.12; leading a scenario exercise: *P*>.99).

## Discussion

### Principal Findings

To our knowledge, this study is the first to report on the development of instructor development workshops for ALS training courses that were conducted in a virtual space with the goal of measuring learning effectiveness. We performed this study by using a commercially available VR collaboration service in combination with several publicly available applications. This workshop could be held safely and remotely during the COVID-19 pandemic.

A total of 5 certified instructor trainers evaluated the workshop trainees’ reports. Since a certain degree of agreement among the raters was observed, a simple average was used as the score. Workshop trainee report scores did not differ between the VR and face-to-face courses.

For the VR group, the median VR-like score was 0, demonstrating that the instructor trainers could not determine whether the VR group was in fact receiving face-to-face instruction or VR instruction. However, the instructor trainers rated the face-to-face group as more “face-to-face–like” than the VR group, and this finding was statistically significant (*P*=.03). This result may have been due to including words specific to the face-to-face course in the reports, such as words related to the performance of defibrillation and the use of a simulator. In other words, the VR course does not adequately reproduce the procedure; therefore, the evaluation may not have been conducted accurately. In the instructor development workshop, the subject matter was how to teach practical skills by using relatively simple skills that can be reproduced in a VR environment, and conducting instructor training in a virtual space was found to have the same learning effect as that of the face-to-face course. However, it is necessary to fully examine whether a VR environment can be used to teach more complex techniques in the future.

The textual analysis of the reports showed that the face-to-face group was characterized by words related to specific actions, while the VR group was characterized by words related to images and creative thinking. In a virtual space, a sense of immersion drives active engagement [16] and increases empathy [17]. It is possible that certain aspects, such as initiative, cohabitation, and imagination, also made a strong impression on the participants in this study. These and similar findings could lead to the discovery of new strengths within VR education.

Based on the questionnaire results of this study, we received many positive responses regarding VR training. However, we believe that many participants found the skills instruction component difficult to implement because the practical considerations for using equipment and tools in a virtual space differed from those in reality [1,8,9]. On the other hand, many participants considered performance evaluation and feedback to be well suited to VR because they were able to repeatedly view the 360° video in order to check evaluation points and provide feedback [18,19]. This finding is supported by the fact that all instructor trainers who were familiar with the conventional face-to-face course responded with a full score of 5.

There may be differences between an instructor development workshop that is conducted in a virtual space and a face-to-face workshop, such as differences in the reproducibility of practical skills [1,8,9]. However, through this study, we were able to confirm that it is possible to hold an entire instructor development workshop for an ALS training course in a virtual space while maintaining the intended educational effect. Since the competencies to be acquired in the instructor development workshop are not practical skills but are ways of teaching practical skills, we believe that learning effectiveness and satisfaction can be enhanced by simple role-plays. The practical skills that can be reproduced in a VR environment and via avatars, such as chest compression and safe electroshock administration, are appropriate as role-play subjects.

As we held the course in an entirely virtual space, participants did not need to gather in person at all. This is an advantage not only in special situations such as the COVID-19 pandemic but also in encouraging the participation of a wide range of students and instructor trainers who have traditionally had difficulties with attending courses due to geographical constraints and other limitations. Thus, workshops that are held in virtual spaces will undoubtedly contribute to the development and recruitment of high-quality instructors.

Since there are no physical constraints in a VR space, there is no need to consider the limitations on the number of participants when securing a venue, which is an issue in face-to-face training. However, in order for a large number of participants to gather in a VR space, each participant must prepare VR equipment, and it is necessary to consider the initial investment in the necessary equipment and acceptable costs. Various VR equipment and applications exist, but they often lack functions for mutual communication and compatibility. A common platform that allows for interactions in a VR space through the use of multiple pieces of equipment and applications may be a solution to this problem.

Students who enroll in a VR course may be positively predisposed toward VR when they are able to indicate their participation after knowing in advance that they are enrolling in a VR course. However, due to the COVID-19 pandemic, a workshop that we offered was rescheduled as a VR course, and the previously enrolled participants were only made aware of this after receiving a call from our administration. Moreover, there was no choice provided to the prospective face-to-face workshop participants. Therefore, bias was considered minimal in this regard.

### Conclusion

This study evaluated instructor development workshops for ALS training courses that were held in a fully virtual space. The learning effect of the VR workshop was the same as that of the face-to-face training workshop. Thus, our findings encourage the implementation of virtual training courses for the effective teaching of ALS skills and may be generalizable to other VR contexts, such as VR medical education, VR training during the COVID-19 pandemic, and other settings that may be amenable to VR-based instruction.

## Abbreviations

ALS: advanced life support
ICLS: immediate cardiac life support
JAAM: Japanese Association of Acute Medicine
VR: virtual reality
